# Treatment of dyslipidemia with lovastatin and ezetimibe in an adolescent with cholesterol ester storage disease

**DOI:** 10.1186/1476-511X-4-26

**Published:** 2005-10-28

**Authors:** Venu T Tadiboyina, Dora M Liu, Brooke A Miskie, Jian Wang, Robert A Hegele

**Affiliations:** 1 Department of Medicine, Schulich School of Medicine and Dentistry, University of Western Ontario, London, ON, N6A 5C1, Canada; 2 Vascular Biology Group and Blackburn Cardiovascular Genetics, Laboratory, Robarts Research Institute, London, ON, N6A 5K8, Canada

## Abstract

**Background:**

Cholesterol ester storage disease (CESD) is an autosomal recessive illness that results from mutations in the *LIPA *gene encoding lysosomal acid lipase. CESD patients present in childhood with hepatomegaly and dyslipidemia characterized by elevated total and low-density lipoprotein cholesterol (LDL-C), with elevated triglycerides and depressed high-density lipoprotein cholesterol (HDL-C). Usual treatment includes a low fat diet and a statin drug.

**Results:**

In an 18-year old with CESD, we documented compound heterozygosity for two *LIPA *mutations: a novel frameshift nonsense mutation and a deletion of exon 8. The patient had been treated with escalating doses of lovastatin for ~80 months, with ~15% decline in mean LDL-C. The addition of ezetimibe 10 mg to lovastatin 40 mg resulted in an additional ~16% decline in mean LDL-C.

**Conclusion:**

These preliminary anecdotal findings in a CESD patient with novel *LIPA *mutations support the longer term safety of statins in an adolescent patient and provide new data about the potential efficacy and tolerability of ezetimibe in this patient group.

## Background

Cholesteryl ester storage disease (CESD; MIM 278000) is an autosomal recessive disorder caused by a deficiency of lysosomal acid lipase (LAL; acid cholesteryl hydrolase; EC 3.1.1.13). LAL is responsible for the intralysosomal hydrolysis of cholesteryl esters (CE) and triglycerides (TG) [1]. Patients with CESD present in childhood with hepatomegaly, hypercholesterolemia and hypertriglyceridemia; most are diagnosed by age 20 [1]. Reduced LAL activity is detectable in peripheral blood leukocytes, cultured skin fibroblasts and liver homogenates [1]. More recently mutational screening of the human LAL gene (*LIPA*) [2-4] has been used for diagnosis. Wolman disease (WD; MIM 278000) also results from mutations in *LIPA*. WD is characterized by early death (usually before age 6 months) and widespread intracellular storage of both CE and TG, mainly in liver, adrenal glands and intestine [1]. *In vitro *catalytic activity was decreased ~200-fold in WD fibroblasts, but only ~50-fold in CESD fibroblasts [5], showing correlation with the differences in phenotypic severity [6,7].

Defective LAL activity results in decreased free intracellular cholesterol [6,7]. Because intracellular free cholesterol normally inhibits 3-hydroxy-3-methylglutaryl-CoA (HMG-CoA) reductase, cholesterol biosynthesis is increased in CESD patients. Thus, pharmacological inhibition of HMG-CoA reductase with statins would seem to be a reasonable approach to restrain the increased cholesterol biosynthesis in CESD. While the plasma lipoprotein response to statins among CESD patients has been variable [8-14], these drugs are considered to be the anti-dyslipidemia agents of choice in CESD.

Ezetimibe is a novel type of lipid-lowering medication that prevents the absorption of cholesterol and plant sterols at the small intestinal brush border by interfering with the activity of the *NPC1L1 *receptor [15-18]. Ezetimibe has been used in adult hypercholesterolemic patients either as monotherapy [19] or in combination with statins [20-25]. The rates of myopathy and serum transaminase elevations in ezetimibe-treated patients appeared to be comparable to those in placebo-treated patients [19-25]. We now present our experience with the combination of lovastatin and ezetimibe treatment in an 18 year old male with CESD.

## Results

### Patient history

A three-year old boy presented to his paediatrician for assessment of a pruritic abdominal rash. His birth and infancy had been unremarkable, with normal growth and development. There was no consanguinity; both parents and two older sisters were all healthy. At age 3, hepatosplenomegaly was noted on abdominal examination and was confirmed by ultrasound. No diagnosis was made and he was monitored periodically. At age 8, he was admitted to the hospital with gastroenteritis. Light microscopy of a liver biopsy showed increased intracytoplasmic glycogen and small lipid droplets in hepatocytes. Electron microscopy showed membrane-bound lipid droplets with small electron dense granules. A working diagnosis of glycogen storage disease type III (DeBrancher disease) was made, but skin fibroblast Debrancher activity was normal.

**Figure 1:**
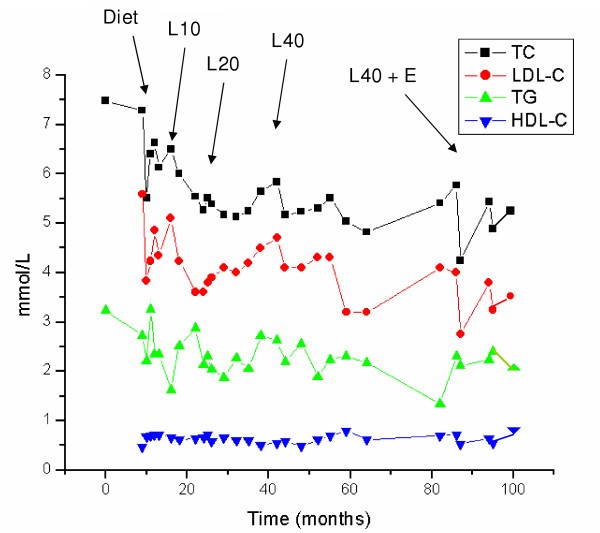
**Plasma lipoprotein responses to treatment**. The graph shows plasma lipoproteins measured serially in the proband over ~96 months. Abbreviations: TC, total cholesterol; LDL-C, low-density lipoprotein cholesterol; TG, triglycerides; HDL-C, high-density lipoprotein cholesterol; Diet, fat restricted to 30% of total calories, L10, L20 and L40 for lovastatin 10, 20 and 40 mg daily, respectively; E, ezetimibe 10 mg daily.

At age 10, hepatomegaly persisted and a second liver biopsy was taken. Light microscopy showed altered lobular architecture of the hepatic parenchyma with distended hepatocytes containing cytoplasmic granules and vacuoles with mild periportal fibrosis. Fibroblast acid lipase activity was found to be 7% of normal, confirming the diagnosis of CESD. Plasma concentrations of total cholesterol (TC), triglycerides (TG), low-density lipoprotein cholesterol (LDL-C) were each above the 95^th ^percentile for age and sex at 7.49, 3.23 and 5.59 mmol/L, respectively, while plasma high-density lipoprotein cholesterol (HDL-C) was below the 5^th ^percentile at 0.46 mmol/L; he had combined hyperlipidemia (hypercholesterolemia, hypertriglyceridemia, hypoalphalipoproteinemia and hyperbetalipoproteinemia). After 12 months, a low fat diet was started (Figure 1).

After 6 months of diet alone, lovastatin 10 mg daily was added. Because of rising plasma concentrations of TC and LDL-C, the dose of lovastatin was increased to 20 mg after ~22 months and increased again to 40 mg after a further ~8 months. Ezetimibe 10 mg per day was added after a further ~40 months and the combination of lovastatin 40 mg and ezetimibe 10 mg daily has continued for 12 months. Serum asparagine transaminase (AST) and creatine kinase (CK) were measured concurrently with the lipoproteins.

The proband's lipoprotein profile since age 10 is summarized in Figure 1. Mean ± standard deviation (SD) lipoprotein concentrations on each phase of treatment were determined from a minimum of three values. From baseline concentrations, the diet was associated with a 5.3% decrease in TC (6.67 ± 0.79 to 6.32 ± 0.26 mmol/L), a 27.3% decrease in TG (2.75 ± 0.48 to 1.99 ± 0.50 mmol/L), a 2.2% increase in LDL-C (4.63 ± 0.77 to 4.73 ± 0.53 mmol/L), a 7.8% increase in HDL-C (0.64 ± 0.12 to 0.69 ± 0.04 mmol/L), and a decrease in TC:HDL-C ratio of 11.5% (10.4 ± 0.3 to 9.2 ± 0.5). When pooled data over ~70 months from all 19 determinations on lovastatin were compared with diet alone, statin treatment was associated with a further 13.5% decrease in TC (to 5.47 ± 0.42 mmol/L), a 12.6% increase in TG (to 2.24 ± 0.36 mmol/L), a 15.9% decrease in LDL-C (to 3.98 ± 0.41 mmol/L), an 8.7% decrease in HDL-C (to 0.63 ± 0.08 mmol/L), and a 5.5% decrease in TC:HDL-C ratio (to 8.7 ± 0.2). Finally, when pooled data over 12 months from all four determinations on ezetimibe plus lovastatin were compared with lovastatin monotherapy, the drug combination was associated with a further 9.4% decrease in TC (to 4.96 ± 0.60 mmol/L), a 3.1% decrease in TG (to 2.17 ± 0.20 mmol/L), an 15.8% decrease in LDL-C (to 3.35 ± 0.46 mmol/L), no change in HDL-C (0.63 ± 0.08 mmol/L), and a 9.1% decrease in TC:HDL-C ratio (to 7.9 ± 0.3). Unpaired t-tests showed that the TC and LDL-C concentrations were significantly different for the period with lovastatin monotherapy compared to the period with combination therapy (P < 0.05). Also, there were no deviations of plasma CK and AST above the upper limit of normal for any treatment period. Finally, liver and spleen size evaluated clinically were reduced compared to baseline over the treatment period with statin and then later statin plus ezetimibe; specifically, while the liver edge was palpable 5 cm below the right costal margin before drug treatment it was not palpable at the most recent clinical assessment.

### Molecular genetic studies

**Figure 2:**
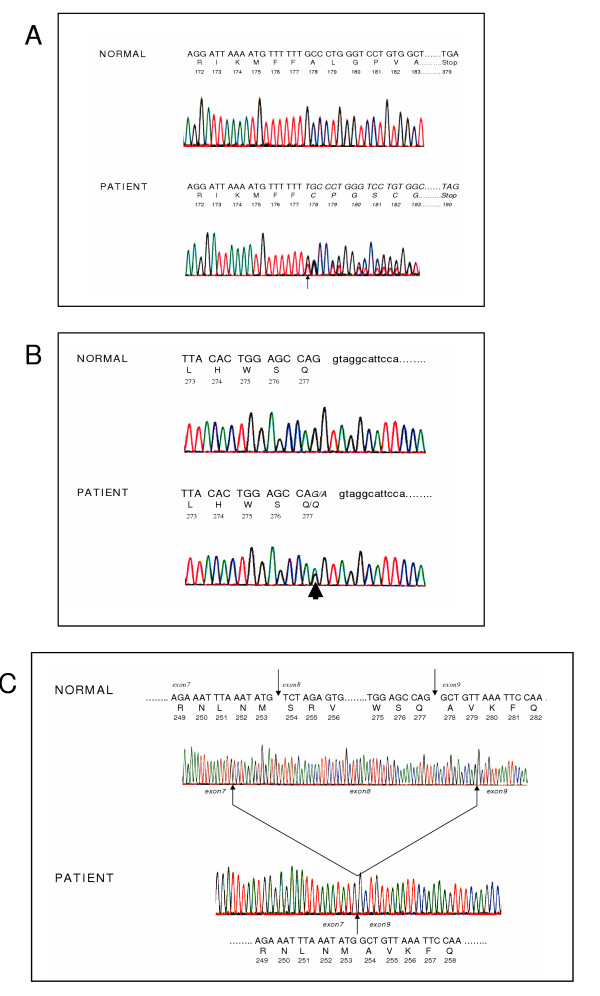
**Nucleotide sequence analysis of *LIPA***. The nucleotide sequences, codon numbers, single letter amino acid codes for the deduced protein sequence are shown in each panel. Panel A shows normal *LIPA *genomic sequence above and the sequence from the proband's genomic DNA below. The inserted nucleotide is indicated by the arrow and the shifted reading frame is suggested by the presence of two peaks at each position following the insertion. Panel B shows normal *LIPA *genomic sequence above and the sequence from the proband's genomic DNA below, with abnormal sequence italicized. The single base nonsense mutation is indicated by the arrow. Panel C shows normal *LIPA *cDNA sequence from a single copy cloned source derived from a normal individual, spanning part of exon 7, all of exon 8 and then part of exon 9. The lower part of the panel shows the cDNA sequence for one of the proband's alleles, in which exon 8 has been deleted in frame. This confirms that the mutation at the intron-exon boundary of exon 8 affected RNA splicing.

Genomic DNA sequencing of the *LIPA *gene revealed that the proband had two mutations (Figure 2). The first was a T insertion in exon 6 at codon 178 that shifted the reading frame (Figure 2A) and caused a premature termination at codon 190 (FS A178-X190). The second was G>A change at the last nucleotide of exon 8 (Q277), which resulted in a silent mutation at the amino acid level (Figure 2B). The patient was heterozygous for both mutations. In order to determine the chromosomal phase of the two *LIPA *mutations, sequencing of exon 6 and exon 8 from the proband's mother's genomic DNA revealed that she was a simple heterozygote for the frameshift mutation, confirming the that two mutations in the proband were on different chromosomes. Reverse transcriptase PCR amplification of *LIPA *from the proband, followed by sequence analysis of the partial cDNA spanning exon 5 through exon 10 revealed an abnormal sequence in which the entire exon 8 had been deleted (Figure 2C).

## Discussion

The untreated lipoprotein profile of our CESD patient revealed not only a combined hypercholesterolemia and hypertriglyceridemia, but also a severe hypoalphalipoproteinemia, indicating that mutations in *LIPA *are a rare genetic cause of complex dyslipidemia. Of course, this is in the context of numerous systemic abnormalities, specifically hepatomegaly. We also showed improvement of the plasma lipoprotein profile with low fat diet, with further improvement with statin monotherapy and even further improvement with the addition of ezetimibe in combination with the statin.

Lovastatin has been shown to be safe and effective in treating hypercholesterolemia over the long term in adults [26,27]. The ~80 month treatment period for our proband was among the longest time spans for any of our young patients with respect to duration of statin therapy. Over this period, the patient's hepatomegaly improved clinically and the AST and CK have remained stable. The addition of ezetimibe was associated with further improvement of plasma lipoproteins, and was also well tolerated in combination with statin treatment.

Statins have previously been successfully used in adolescents with CESD with some variability in reported efficacy [8-14]. This may be explained by genetic heterogeneity in response to lovastatin or by underlying differences in the factors responsible for the hyperlipidemia [3] Statins block the conversion of 3-hydroxymethylglutaryl-coenzyme A (HMGCoA) to mevalonate, a rate limiting step in cholesterol biosynthesis. This results in an increase in the number and activity of LDL receptors on the hepatocyte membrane, and the rate of LDL catabolism increases. In patients with CESD, the increased activity of the LDL receptors theoretically could lead to increased accumulation of cholesteryl esters in the liver [4]. However, Ginsberg *et al*. [2] showed no change in hepatic cholesteryl ester accumulation after 8 months of lovastatin 40 mg daily in a 9 year old girl with CESD. Furthermore, our patient had reduced hepatomegaly clinically, suggesting that cholesteryl ester accumulation in the liver was unlikely.

Our findings also suggest that ezetimibe may be a useful treatment in patients with CESD. Ezetimibe interferes with the normal function of the *NPC1L1 *gene product, which regulates sterol absorption in the small intestine [15-17]. This is thought to result in depletion of hepatic cholesterol and upregulation of hepatic LDL receptors. The mean plasma LDL-C reduction seen with ezetimibe is ~20%, and this has been remarkably consistent across patient subgroups defined by age, gender, ethnic background and concomitant use of other lipid regulating agents, such as statin drugs [20-25]. Inter-individual genetic variation may also play a role in the response to ezetimibe; for instance, a subset of individuals with a particular *NPC1L1 *haplotype appears to have a larger plasma LDL-C response [28]. Combination therapy for hypercholesterolemia may allow more patients to achieve target plasma TC and LDL-C goals. We observed that coadministration of ezetimibe with lovastatin resulted in reduction in plasma LDL-C concentration of 16% compared to lovastatin alone. Ezetimibe appeared to be well tolerated by our patient and there were no reported adverse effects. Ezetimibe added to lovastatin did not result in an increase in muscle enzymes. In fact, serum CK (mean ± SD) actually decreased from 185 ± 156 with statin monotherapy to 120 ± 68 during combination treatment with the statin and ezetimibe. AST levels never exceeded the upper limit of normal at any time. The complementary mechanism of action of ezetimibe and statins may offer a new treatment alternative for dyslipidemia management in CESD patients.

## Methods

### Genomic DNA analysis

Genomic DNA was isolated from whole blood obtained from patients (Puregene, GentraSystem, Minneapolis, MN). The entire coding region and intron-exon boundaries of LAL gene were amplified using custom primer pairs shown in table 1. PCR amplifications were carried out in a 50 μl mixture containing 32 pmole of each primer, 0.2 mM of each dATP, dCTP, dGTP and dTTP, 1.5 mM MgCl_2_, 50 mM KCl, 20 mM Tris-HCl (pH 8.4), 2.5 units of Taq platinum DNA polymerase (Invitrogen, Mississauga, ON). 30 cycles were performed consisting of denaturation at 94°C, annealing at 56°C and extension at 72°C for 30 s each, followed by extension for 10 min at 72°C. PCR products were purified from agarose (QIAQuick Gel Extraction Kit, Qiagen, Mississauga, ON). Direct DNA sequence results were analyzed with an ABI automated sequencer 3730 and were read with ABI Sequence Navigator software (both from PE Biosystems, Mississauga, ON).

**Table 1:** Primers used to amplify coding regions of *LIPA*

Exon	Primer sequences
1	Forward: 5' AGC GCT AAA CAG CTT GCT AG
	Reverse: 5' CTT GCT GAA GGC ACC AGC
	
2	Forward: 5' GGC TGG AGT CAT TTG TTT CA
	Reverse: 5' AGA ATC ACT TGA GCC CCT GA
	
3	Forward: 5' GCC TGG AGA ACA TAG TTT ATC TGC
	Reverse: 5' TTA GAT GAC TCT TGT CCT TAC TTC
	
4	Forward: 5' ATG TGA GTA CAT CAC TAT GTC
	Reverse: 5' CTC ATA CAA CTT CAG AGT TAC
	
5	Forward: 5' TTC CCA GCT GTG TTT AGT TTG TG
	Reverse: 5' GAC TAA ATG TTA CCA ACA TTC C
	
6	Forward: 5' GTG TTA GGG CAC ACG GAA GT
	Reverse: 5' GTG TGC AGG AAA CGA CAG G
	
7	Forward: 5' GCA TCC TGA TTT GAT GTC CA
	Reverse: 5' CAT AAG AAG GTG ACC ACA GTC AG
	
8	Forward: 5' TGG CTC TAG TTT TTA GTG CTT TGA
	Reverse: 5' GGA CTC TGG GGA AGA AAA CC
	
9	Forward: 5' TTC TGT GTC AGG TGG TAG CTG
	Reverse: 5' TGG ACT GAT GGA AAA CAA ACA
	
10	Forward: 5' CTC CAC AGC TAG TGG CGA TT
	Reverse: 5' CAC ACA ATT CTT TGG GCC TAT

### Reverse transcriptase polymerase chain reaction and cDNA sequence analysis

Total RNA was isolated from the proband using PAXgene Blood RNA Tube and Blood RNA Kit (Qiagen, Mississauga, ON). 2.5 ml blood was used for RNA isolation according to manufacturer's instruction. 100 ng of total RNA was used for first strand cDNA synthesis (SuperScript First Strand Synthesis System, Invitrogen, Mississauga, ON). 2 μl of first strand reaction was used for amplify partial cDNA sequence of the *LIPA *gene spanning exon 5 to 10 with primer pair 5' AAT ATG ACC TAC CAG CTT CCA, 3' GTA AGC AAA CAC ATT TTC ACA. PCR products were gel purified, sequenced and read as described above.

## Authors' contributions

VT: data analysis, manuscript preparation and approval

DML: data analysis, manuscript preparation, manuscript approval

BAM: data collection, patient interaction, manuscript approval

JW: sequencing, data analysis, editing, manuscript approval

RAH: experimental design, manuscript preparation and approval
